# A prenatal case misunderstood as specimen confusion: 46,XY/46,XY chimerism

**DOI:** 10.1186/s12884-024-06321-5

**Published:** 2024-02-12

**Authors:** Lin Chen, Li Wang, Yang Zeng, Daishu Yin, Feng Tang, Dan Xie, Hongmei Zhu, Lingping Li, Jing Wang

**Affiliations:** 1 Department of Medical Genetics / Prenatal Diagnostic Center, West China Second University Hospital, Sichuan University, Block 3 No. 20, Ren Min Nan Road, Wuhou District, Chengdu, 610041 China; 2 Key Laboratory of Birth Defects and Related Diseases of Women and Children, Sichuan University, Ministry of Education, Chengdu, 610041 China

**Keywords:** Chimerism, Short tandem repeat, Prenatal diagnosis

## Abstract

Chimerism results from the fusion of two zygotes in a single embryo, whereas mosaicism results from mitotic errors in a single zygote. True human chimerism is rare, with fewer than 100 cases reported in the literature. Here, we report a case in which the fetus was identified as having tetragametic chimerism based on short tandem repeat - polymerase chain reaction analysis of the family observed during amniocentesis for advanced maternal age. The chimerism occurred via the fertilization of two ova by two spermatozoa, followed by the fusion of early embryos. The genotypes of the two amniotic fluid samples obtained successively by one puncture were completely different, and the sex chromosomes were XY. Karyotyping and copy number variation sequencing showed no abnormalities. The fetus was delivered at term and the phenotype of the newborn was normal.

## Introduction

Amniocentesis is the most commonly used invasive technique for fetal diagnosis. Before conducting molecular genetic testing of amniotic fluid samples, maternal cell contamination testing was performed [1]. Polymorphic short tandem repeat (STR) marker analysis has become a commonly used method for human identity testing. STR analysis is the most commonly used method for detecting maternal cell contamination in laboratories and can also be performed on twin samples to determine whether they are homozygotic or dizygotic twins. In addition, selecting STR loci on specific chromosomes for detection may enable faster identification of common fetal aneuploidies [2].

Chimerism is usually restricted to certain tissues and is acquired through therapeutic interventions, such as hematopoietic stem cell transplantation [3, 4]. Acquired chimerism is a common finding, while reports of true human chimerism are rare [5, 6]. True human chimerism results from the fusion of two different zygotes into a single embryo. Gartler et al. first described a case of chimerism in 1962 [7]. Since then, fewer than 100 cases have been reported. Here, we describe a case of 46,XY/46,XY chimerism observed during amniocentesis for advanced maternal age. We initially speculated that this may be due to sample confusion, but ultimately confirmed that it was tetragametic chimerism. The pregnancy resulted in a healthy baby at term.

## Case report

A 34-year-old pregnant woman was referred to our center for amniocentesis at 19 weeks of gestation because of advanced maternal age (35 years on the estimated date of delivery). The patient’s family history was unremarkable. This was the fifth pregnancy, after previous pregnancies resulted in a healthy child, an early voluntary abortion and two spontaneous abortions. Her husband was 34 and in good health. Due to two spontaneous miscarriages, the couple underwent karyotyping of peripheral blood cells 4 years prior, and the results were normal.

**Fig. 1:**
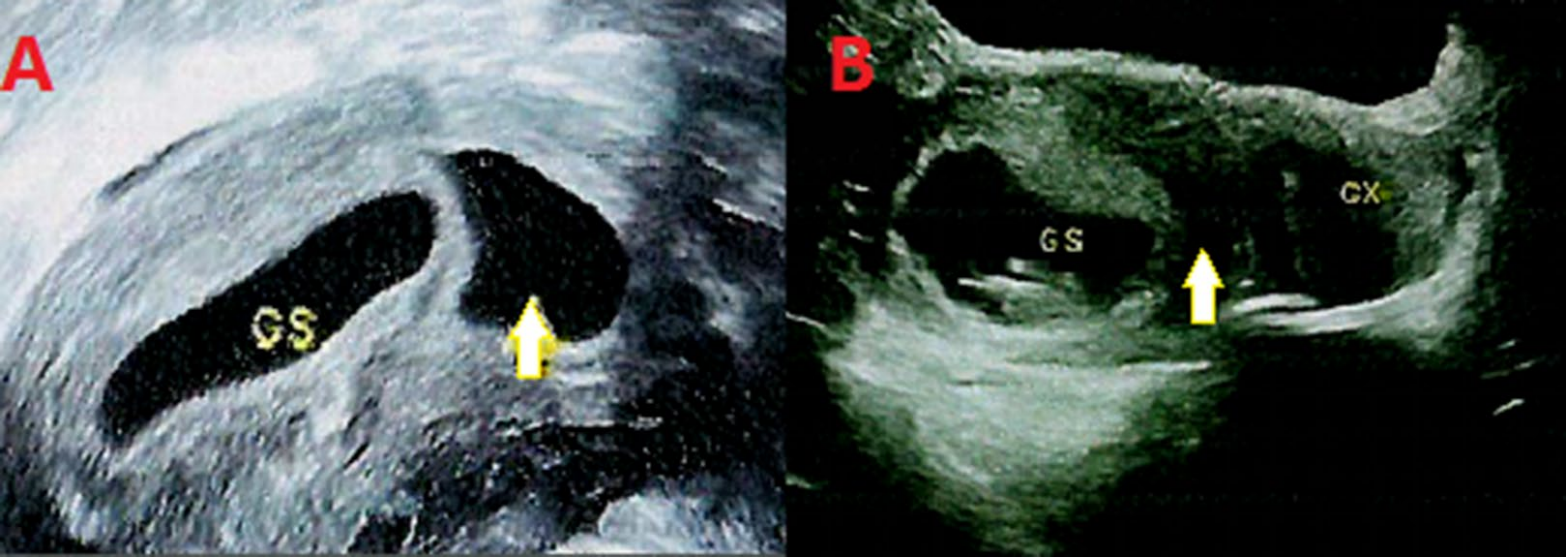
The ultrasound results during early pregnancy. (**A**) Ultrasound image at 7^+2^ weeks gestation. (**B**) Ultrasound image at 9^+2^ weeks gestation. GS: Gestation sac. The area indicated by the yellow arrow represents the echoless zone

This pregnancy was the result of natural conception, and two ultrasounds during early pregnancy indicated a viable embryo and an echoless zone in the uterine cavity, which was suspected to be an aborted pregnancy sac or fluid accumulation. The ultrasound results during early pregnancy are shown in Fig.1. Ultrasound examination at 12^+ 1^ weeks of gestation showed a single live fetus with a normal nuchal translucency value, and the gestation period was consistent with the calculation based on the last menstrual period. Throughout the pregnancy, the woman underwent regular check-ups in the obstetric department as prescribed by her doctor. No clinically significant variation was found in the chromosome karyotype or through copy number variation sequencing (CNVseq) analysis of the amniotic fluid. Multiple ultrasonography results during pregnancy indicated normal fetal development. After a full-term pregnancy, a healthy boy was delivered. The newborn was 50 cm long and weighed 3,300 g. The Apgar score of the newborn was normal and there were no abnormalities in appearance or feeding.

## Materials and methods

According to routine operational specifications, 22 mL of amniotic fluid was extracted and placed in six sterile centrifuge tubes. The puncture did not pass through the placental tissue, and the surgery was completed successfully. The amniotic fluid was light-yellow and transparent. Two tubes with 5 mL samples were subjected to karyotyping, two tubes with 3 mL samples (named prenatal sample 1 and prenatal sample 2) were subjected to multiplex short tandem repeat-polymerase chain reaction (STR-PCR) and CNVseq analyses, and the remaining two tubes were stored at 2–8℃.

Karyotyping after GTG banding was performed according to standard procedures for cultured amniotic fluid cells. DNA was extracted from the amniotic fluid and parental peripheral blood cells using a DNeasy Blood and Tissue Kit (Qiagen, Hilden, Germany), according to the manufacturer’s instructions. STR-PCR was performed using 14 autosomal markers and 6 sex-linked markers (Daan Gene, Guangzhou, China). According to the manufacturer’s instructions, the PCR conditions were as follows: an initial denaturation at 95℃ for 5 min, followed by 25 cycles of 95℃ for 30 s, 58℃ for 40 s, and 72℃ for 50 s, with a final elongation step at 72℃ for 10 min. PCR products were separated using an ABI 3500 Genetic Analyzer (Applied Biosystems, Waltham, MA, USA) and the results were analyzed using GeneMapper Software 5 (Applied Biosystems). DNA libraries were prepared using a Chromosome CNV Detection kit (Berry Genomics, Beijing, China) and subsequently sequenced on a NextSeq500 sequencing platform using a NextSeq500 High Output kit (Illumina, San Diego, CA, USA), according to the manufacturer’s instructions. Based on the resolution of this technology, the pathogenicity of copy number variants (CNVs) > 100 kb was analyzed. The clinical significance of the detected CNVs was interpreted according to the American College of Medical Genetics and Genomics standards and guidelines [8].

## Results

**Fig. 2:**
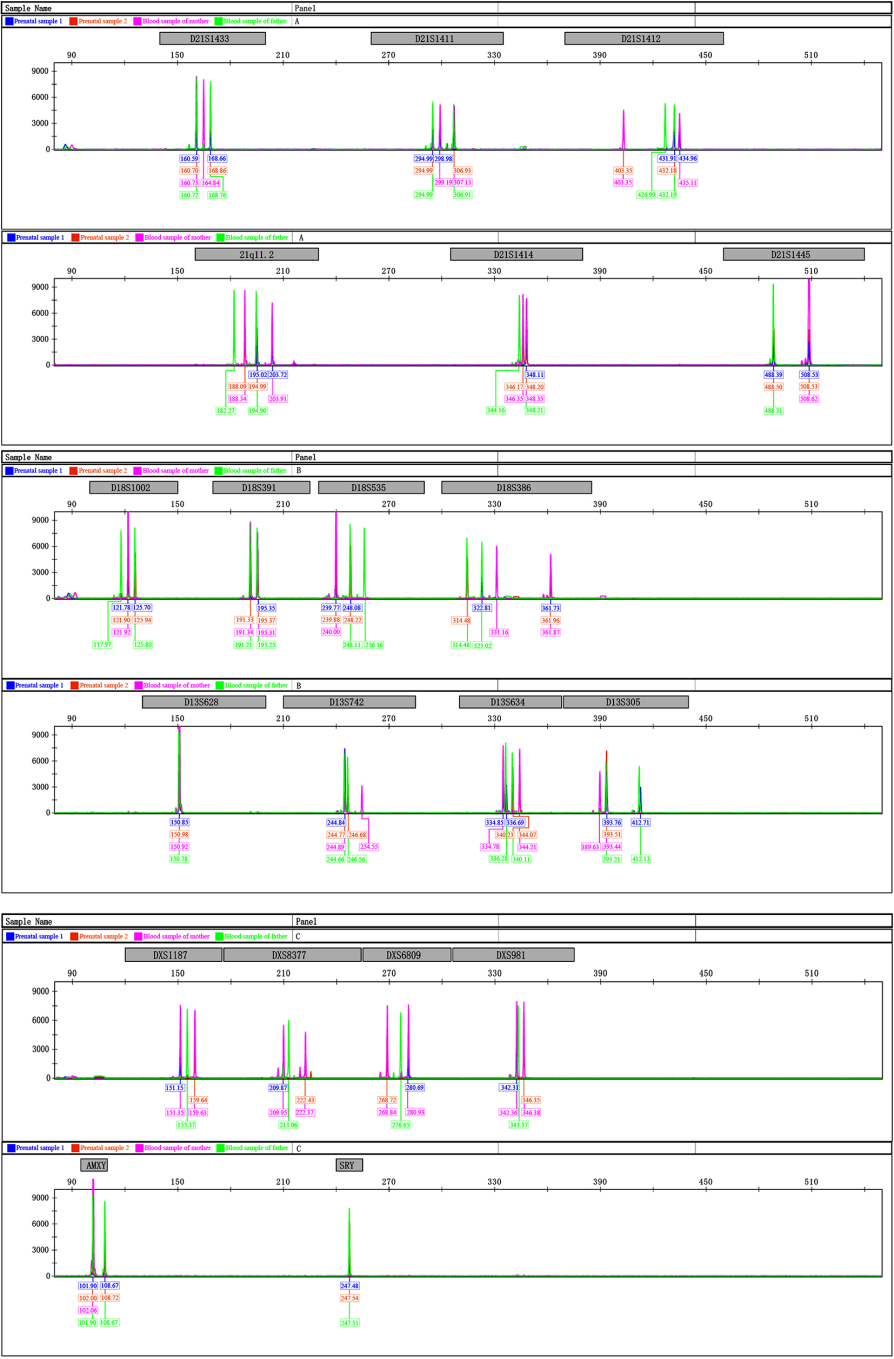
The results of the STR analysis

The karyotype obtained from the amniotic fluid cell samples was 46,XY. No clinically significant CNVs were found in CNVseq analysis, and the copy numbers of the X and Y chromosomes were 1. Prenatal sample 1 showed two alleles each for 11/20 STRs, the other nine STRs had one allele each. However, prenatal sample 2 showed two alleles each for 13/20 STRs, the other seven STRs consisted of one allele. The test results of both samples suggested that they were samples from a single individual, and the STR results of the sex chromosomes showed that they were both XY, with no DNA contamination from another individual. However, some alleles of the STR loci in the two samples were different. By comparing the allele results of the two samples, we found that the peak heights (RFU-relative fluorescence units) of only seven STR loci (D21S1433, D21S1445, D18S1002, D18S535, D13S628, AMXY, and SRY) were consistent between the two samples. The alleles of the remaining 13 STR loci differed, suggesting the two samples were from different individuals. At first, the laboratory staff were concerned that the samples were confusing, and after confirming other samples from this batch, no other suspected confused samples were found. STR-PCR was performed on the remaining two tubes of amniotic fluid samples, and it was found that the alleles at the STR loci of one tube were the same as those of prenatal sample 1, and those of the other tube were the same as those of prenatal sample. 2. Finally, the laboratory staff informed the parents of the fetus to draw blood and perform STR-PCR testing. By comparing the STR loci among the family members, we found that the alleles of both prenatal samples 1 and 2 came from the father or mother, but the two prenatal amniotic fluid samples of this fetus belonged to two completely different sources. The results of the family STR analysis are presented in Table 1; Fig. 2.

**Table 1:** Complete STR-resolution of the fetus and parents

STR	D21S1433	D21S1411	D21S1412	21q11.2	D21S1414	D21S1445	D18S1002	D18S391	D18S535	D18S386
**Prenatal sample 1**	160.59/168.66	294.99/298.98	431.91/434.96	195.02/203.72	348.11	488.39/508.53	121.78/125.70	195.35	239.77/248.08	322.81/361.73
**Prenatal sample 2**	160.70/168.86	294.99/306.93	403.35/432.18	188.09/194.99	346.17/348.20	488.50/508.53	121.90/125.94	191.33/195.37	239.88/248.22	314.48/361.96
**Mother**	160.73/164.84	299.19/307.13	403.35/435.11	188.34/203.91	346.35/348.35	508.62	121.92	191.34/195.31	240.00	331.16/361.87
**Father**	160.72/168.76	294.99/306.91	426.99/432.19	182.27/194.90	344.16/348.21	488.31	117.97/125.80	191.21/195.25	248.11/256.16	314.48/323.02
**STR**	**D13S628**	**D13S742**	**D13S634**	**D13S305**	**DXS1187**	**DXS8377**	**DXS6809**	**DXS981**	**AMXY**	**SRY**
**Prenatal sample 1**	150.85	244.84	334.85/336.69	393.76/412.71	151.15	209.87	280.69	342.31	101.90/108.67	247.48
**Prenatal sample 2**	150.98	244.77/246.68	340.23/344.07	393.51	159.64	222.43	268.72	346.35	102.00/108.72	247.54
**Mother**	150.92	244.89/254.55	334.78/344.21	389.63/393.44	151.35/159.63	209.95/222.37	268.84/280.93	342.36/346.38	102.06	/
**Father**	150.78	244.66/246.56	336.28/340.11	393.21/412.13	155.37	213.06	276.65	343.37	101.90/108.67	247.51

*Abbreviation* STR, short tandem repeat

## Discussion

In our case, a single pregnant woman at 19 weeks of gestation underwent amniocentesis for prenatal diagnosis due to advanced maternal age, and STR analysis indicated that the two prenatal amniotic fluid specimens of the fetus belonged to two completely different individual sources. Seven STR loci with the same allelic information were excluded, and another nine autosomal markers (D21S1411, D21S1412, 21q11.2, D21S1414, D18S391, D18S386, D13S305, D13S742, and D13S634) and four X-linked markers (DXS1187, DXS8377, DXS6809, and DXS981) were analyzed. All STR alleles of the two prenatal amniotic fluid specimens were found either in the father or the mother, and they came from different gametes. This indicated that we detected four gametes in one fetus. Two ultrasounds during early pregnancy indicated a viable embryo and an echoless zone in the uterine cavity, which was suspected to be an aborted pregnancy sac or fluid accumulation. Therefore, after comprehensive analysis, we believe that the fetus has tetragametic chimerism (46,XY/46,XY), which occurred via fertilization of the two ova by two spermatozoa, followed by the fusion of early embryos and the development of an organism with intermingled cell lines.

Chimerism results from the amalgamation of two different zygotes into a single embryo, whereas mosaicism results from mitotic errors in a single zygote. Tetragametic chimerism is rare; however, most cases remain unrecognized. When one of the twins or multiple embryos dies in utero, disappear or get resorbed partially or entirely, with an outcome of a spontaneous reduction of a multi-fetus pregnancy to a singleton pregnancy, it is called vanishing twin syndrome. This phenomenon occurs commonly during the first trimester, and occurs in about half of pregnancies with three or more gestational sacs, and in 36% twin pregnancies. Certain etiological factors are considered to be associated with the loss of the embryo, including advanced maternal age, chromosomal abnormalities in the deceased twin, use of assisted reproductive techniques, genetic and teratogenic factors, etc. [9]. If the surviving embryo absorbs the dead embryonic tissue, it forms chimerism. Chimerism resulting from the fusion of XX and XY embryos is most likely to be discovered, because mixed gonads can result in abnormalities in the external genitalia [10]. Same-sex chimerism is less likely to be detected and only a few cases have been reported in humans [6, 11]. XY/XY chimerism has not been reported. The two embryos of dizygotic twins have their own genetic material and are at independent risk for genetic diseases theoretically. Therefore, if an individual exhibits tetragametic chimerism, genetic testing should be performed on both cell lines whenever possible. Chromosomal abnormalities have been reported in individuals with chimerism, such as 46,XX/47,XY,+21, 46,XY/47,XYY, and 47,XY, + 8/46,XX chimerism [12–15]. In the present study, both prenatal amniotic fluid specimens showed normal and identical sex chromosomes (XY/XY). Therefore, theoretically, the fetus will not have a phenotype associated with chromosomal diseases after birth, and its prognosis should be good. Multiple fetal ultrasounds during pregnancy indicated no abnormalities, and follow-up after birth confirmed that the newborn had no abnormal appearance or abnormal development.

It has been nearly 60 years since the prenatal diagnosis of genetic diseases was first offered. Fetal samples are commonly obtained by chorionic villus sampling (CVS), amniocentesis, or percutaneous umbilical blood sampling (PUBS). Although CVS is usually performed from 10 to 14 weeks of gestation, and prenatal diagnosis results can be obtained earlier, genetic testing of a sample obtained by CVS may yield a false-positive or false-negative result if the fetus and placenta are genetically discordant, as in confined placental mosaicism, which is thought to occur in 1–2% of CVS samples [16, 17]. PUBS carries a higher risk of fetal loss than CVS or amniocentesis, which is mainly reserved for cases in which diagnostic information cannot be obtained through amniocentesis or CVS. Amniocentesis is usually performed after 16 weeks of gestation, is simpler to perform, and has a lower risk of fetal loss than CVS or PUBS [18]. Amniocentesis is the most commonly used invasive technique for fetal diagnosis. The sources of cells in the amniotic fluid include three types of cells derived from the ectoderm, mesoderm, and endoderm. For example, amniotic fluid contains exfoliated epidermal cells from the ectoderm, exfoliated urinary system cells from the mesoderm, and exfoliated digestive tract cells from the endoderm. Although the proportion of cells derived from the three germ layers in the amniotic fluid is uncertain, theoretically, amniotic fluid remains the best sample for the diagnosis of fetal chimeras or mosaics, and its detection results can represent the genetic composition of the fetus more comprehensively. The genetic material of the different tissues and organs in chimeras may be inconsistent. In theory, genetic testing for multiple germ layers is more comprehensive; therefore, amniotic fluid cells are a more suitable specimen for prenatal diagnosis of fetuses. Theoretically, fetal cells originating from different germ layers are well mixed in the amniotic cavity; therefore, the genetic testing results of the samples taken at the same time should be consistent. However, in this study, two amniotic fluid samples taken simultaneously (interval less than 1 min) after one puncture showed completely different genotypes, which is rare and has not yet been reported. We speculated that it may, after the first amniotic fluid sample was extracted, vigorous fetal exercise may have caused a change in the composition of the cell source in the amniotic cavity or that newly shed cells of another genetic origin were obtained during the extraction of the second sample. After analyzing 20 karyotypes of the two tubes of amniotic fluid samples, no polymorphic changes or abnormalities were found; therefore, the source of the cell lines of the two tubes of samples could not be determined.

In conclusion, we report a case in which the fetus was identified as having tetragametic chimerism by family STR-PCR analysis and early ultrasound. This chimerism occurred via the fertilization of two ova by two spermatozoa, followed by the fusion of early embryos. The STR genotypes of the two amniotic fluid samples obtained successively by one puncture were completely different, and the sex chromosomes were XY. Karyotyping and CNVseq test results showed no abnormalities. The fetus was delivered at term and the phenotype of the newborn was normal.

## Data Availability

The data underlying this article are available in the article.
